# Effect of music listening on hypertonia in neurologically impaired patients—systematic review

**DOI:** 10.7717/peerj.8228

**Published:** 2019-12-19

**Authors:** Tamaya Van Criekinge, Kristiaan D’Août, Jonathon O’Brien, Eduardo Coutinho

**Affiliations:** 1 Department of Rehabilitation Sciences and Physiotherapy, REVAKI/MOVANT, Universiteit Antwerpen, Antwerp, Belgium; 2 Department of Musculoskeletal Biology, University of Liverpool, Liverpool, United Kingdom; 3 School of Health Sciences, University of Liverpool, Liverpool, United Kingdom; 4 Applied Music Research Lab, Department of Music, University of Liverpool, Liverpool, United Kingdom

**Keywords:** Hypertonia, Spasticity, Music, Electromyography, Relaxation, Neurology

## Abstract

**Background:**

As music listening is able to induce self-perceived and physiological signs of relaxation, it might be an interesting tool to induce muscle relaxation in patients with hypertonia. To this date effective non-pharmacological rehabilitation strategies to treat hypertonia in neurologically impaired patients are lacking. Therefore the aim is to investigate the effectiveness of music listening on muscle activity and relaxation.

**Methodology:**

The search strategy was performed by the PRISMA guidelines and registered in the PROSPERO database (no. 42019128511). Seven databases were systematically searched until March 2019. Six of the 1,684 studies met the eligibility criteria and were included in this review. Risk of bias was assessed by the PEDro scale. In total 171 patients with a variety of neurological conditions were included assessing hypertonia with both clinicall and biomechanical measures.

**Results:**

The analysis showed that there was a large treatment effect of music listening on muscle performance (SMD 0.96, 95% CI [0.29–1.63], *I*^2^ = 10%, *Z* = 2.82, *p* = 0.005). Music can be used as either background music during rehabilitation (dual-task) or during rest (single-task) and musical preferences seem to play a major role in the observed treatment effect.

**Conclusions:**

Although music listening is able to induce muscle relaxation, several gaps in the available literature were acknowledged. Future research is in need of an accurate and objective assessment of hypertonia.

## Introduction

A common symptom of upper motor neuron syndromes is hypertonia which is defined as an increased muscle tone, while lower motor neuron syndromes are related to hypotonia or decreased muscle tone. Two types of hypertonia can be distinguished: pyramidal or extrapyramidal hypertonia. First, pyramidal hypertonia also known as spasticity is defined as “a velocity-dependent increase in muscle tone as a result hyper excitability of the stretch reflex” (Lance, 1980) and is mostly pronounced during voluntary movements. Second, extrapyramidal hypertonia or rigidity, is not speed-dependent and is therefore also present during slow passive movement (Singer et al., 2010). As inhibition of supraspinal inputs is impaired, antagonistic muscles become less effective in responding to agonist muscle contractions (Gracies, 2005a; Gracies, 2005b; Singer et al., 2010). This can either lead to prolonged shortening of a muscle as the joint is fixed in a certain position (a.k.a. contracture) (Gracies, 2005b) or it can lead to simultaneous co-contraction of agonist and antagonist muscles which results in an immediate resistance of the movement (Singer et al., 2010). Contractions are the result of decreased muscle mass and sarcomeres and an accumulation of connective tissue and fat (Gracies, 2005a; Gracies, 2005b).

Spasticity-related interventions mainly consist of pharmacological and/or physio- and occupational therapeutic approaches (Katz, 1988; Nair & Marsden, 2014; Rekand, 2010; Thompson et al., 2005). Pharmacological treatment consists of oral anti-spasticity drugs, botulinum toxin injections or intrathecal baclofen pump. Physio- and occupational therapeutic management consists of stretching/splinting and positioning as preventive care for contractures and to preserve comfort. Subsequently, exercise therapy mainly consists of reducing abnormal sensory inputs and providing adequate muscle length. However, high-quality evidence for supporting the effectiveness of these rehabilitation techniques is lacking (Amatya et al., 2013; Khan et al., 2017; Nair & Marsden, 2014). A low quality for evidence was found for rehabilitation programs, electrical stimulation, physical activity programs, vibration therapy, stretching and passive movement in neurological patients (Khan et al., 2017). There is a clear need for effective non-pharmacological rehabilitation strategies to treat spasticity. In addition, there is only a limited amount of evidence regarding the treatment of rigidity and this relates mainly to the general management of Parkinson’s disease (Diamond & Jankovic, 2006; Guttman, Kish & Furukawa, 2003).

Music interventions are gaining popularity in rehabilitation programs and are able to induce self-perceived and physiological outcomes of relaxation in healthy adults (Largo-Wight, O’Hara & Chen, 2016; Madson & Silverman, 2010; Staum & Brotons, 2000; Stratton & Zalanowski, 1984), hospitalized (Krout, 2001; Sand-Jecklin & Emerson, 2010), burn (Tan et al., 2010), psychiatric (Thaut, 1989), oncology (Cooper & Foster, 2008) and neurological patients (Sihvonen et al., 2017). Music as an intervention is mostly empowered by its patient-centered character, it is a pleasurable intervention which has the secondary benefit of eliciting arousal and emotional responses. Listening to music activates cortical and paralimbic areas related to neural systems of reward and emotion (Blood & Zatorre, 2001), which makes music an intervention which can be rewarding and motivating and at the same time regulate emotions, arousal and cognitive functions (Sarkamo, 2018). So music interventions might be a good multi-modal treatment option for inducing muscle relaxation in neurological patients with hypertonia, and might lead to a better therapy compliance by its enjoyable character.

Music interventions can be categorized into two types of interventions—passive or receptive (i.e., listening to music) compared to active (e.g., producing music or have an active role during therapy) (Tang & Vezeau, 2010). This study is mainly focused on the effects of passive interventions as active music interventions (e.g., playing an instrument) are not able to distinguish the cause of the observed changes in motor behavior, which can either be due to the music heard or other behaviors associated with the therapy received. Music listening does not include sound-based interventions which are merely based on rhythmical sound sequences or cues, also known as Rhythmic Auditory Stimulation (RAS) or auditory cueing. This treatment does not elicit similar changes in performance and secondary beneficial effects on mood and arousal (Styns et al., 2007; Thaut, Rathbun & Miller, 1997; Wittwer, Webster & Hill, 2013).

The aim of this study is to investigate if music listening interventions (MLI) are an effective tool to decrease muscle tension in neurologically impaired patients suffering from hypertonia. As listening to music is able to induce general relaxation in several patient populations, we hypothesize that relaxation on muscular level will be present.

## Survey Methodology

### Protocol and registration

This review was conducted according to the Preferred Reporting Items for Systematic Review and Meta-Analysis Statement (PRISMA). The checklist can be found as Supplementary Material 1 (Moher et al., 2009). The study was registered in the PROSPERO database (CRD: no. 42019128511).

### Eligibility criteria

Studies were included if they met the following criteria:

(1) The study population included patients suffering from a neurological disorder which could result in hypertonia e.g., stroke, cerebral palsy, Parkinson’s disease, spinal cord injury, multiple sclerosis, etc.
(2) Interventions had to include MLI
(3) Muscle tone or activity had to be assessed after intervention. Both clinical and biomechanical analysis of muscle performance were included, e.g., modified Ashworth scale, motor assessment scale, electromyography (EMG), etc.
(4) Studies had to be written in English, Dutch, German, French or Spanish.
(5) Study design was a (randomized) clinical trial.

Studies were excluded using the following criteria:

(1) Music listening involving other sound-based interventions, e.g., RAS or auditory cueuing;
(2) Studies pertaining to active music interventions (i.e., singing, playing rhythms on musical instruments).

### Information sources

A systematic search strategy was conducted using the electronic databases of PubMed, Web of Science, Cochrane Library, Science Direct, Scopus, ResearchGate and the Physiotherapy Evidence Database (PEDro). A combination of free text words (spastic, spasticity, muscle tone, hypertonicity, hypertonus, hypertonic, hypertonia and muscle activity) and Medical Subject Headings were used (music, muscle spasticity, muscle hypertonia and electromyography) after careful consideration from all authors. Discrepancies were discussed by all authors and resolved when a majority agreed. The details of the final search strategy, performed in March 2019, can be found as Supplementary Material 2.

### Study screening

The screening procedure was performed by three independent researchers (Van Criekinge, O’Brien, and D’Août). To collect potentially relevant studies, eligibility was screened based on title and abstract. Full texts were retrieved and evaluated based on the a-priori provided inclusion and exclusion criteria. Afterwards full texts were gathered and evaluated on the previously set inclusion criteria. Reference lists were manually screened to identify additional relevant studies. Discrepancies were discussed with a third independent person (either O’Brien, D’Août, depending on the allocated studies).

### Assessment of quality

The risk of bias was assessed by two independent reviewers (Van Criekinge and Coutinho) by using the PEDro scale for randomized controlled trials (Maher et al., 2003). In case of uncertainty at any point during the scoring process, consensus was sought by a third reviewer (O’Brien). The PEDro scale assesses eleven items such as random allocation of the subjects, concealed allocation and blinding of therapists and assessors (Maher et al., 2003). The total PEDro score can be divided into three sections; high quality = PEDro score 6–10, fair quality = PEDro score 4–5 and poor quality = PEDro score ≤3.

### Data extraction and analysis

Extracted data consisted of subject characteristics (age, gender, pathology), outcome measures, type of music or apparatus, intervention and results (see Table 1). Results were mostly described as a difference between groups based on intervention.

**Table 1:** Data-extraction of the included studies.

**Author (Year)**	**Participants**		**Music**	**Outcome measure**	**Intervention**		**Results**	
	**Condition (*n*, m/w)**	**Age(years, SD)**			**Experimental**	**Control**	**Experimental**	**Control**
Ben-Pazi et al. (2018)	CP (*n* = 18, 13/5)	7.5 (4.1)	4–5 audio tracks: music/nature sounds-headphones	CCHQ (/7)	Standardized sound freq/amp: gamma range (*n* = 9)	Only sound/music listening (*n* = 9)	Pre: 3.6 Post: 2.1	Pre: 3.26 Post: 3.23
					30 min, 4x/week, 4 weeks		*p* = 0.002	
Jeba & Joshi (2016)	Stroke (*n* = 19)	53.30 (9.18) 52.11 (9.98)	Eastern classical instrumental piece—headphones	MAS (/5)	Receptive listening to relaxing music prior to conventional therapy (*n* = 10)	Only conventional therapy: inhibitory/facilitatory techniques, weight bearing, stretch, rhythmic rotation (*n* = 9)	**Shoulder abduction (*p* = 0.22)** Pre: 1 (#2), 1+ (#7), 2 (#1) Post: 1 (#6), 1+ (#2), 2 (#2) **Elbow flexors (*p* = 0.02)** Pre: 1 (#4), 1+ (#2), 2 (#3), 3 (#1) Post: 0 (#1), 1 (#5), 1+ (#3), 2 (#1) **Forearm pronators (*p* = 0.018)** Pre: 1 (#2), 1+ (#3), 2 (#5) Post: 0 (#1), 1 (#6), 1+ (#3) **Hip adductors (*p* = 0.04)** Pre: 0 (#1), 1 (#3), 1+ (#6) Post: 0 (#2), 1 (#7), 1+ (#1) **Knee extensors (*p* = 0.04)** Pre: 0 (#1), 1 (#7), 1+ (#1), 3 (#1) Post: 0 (#4), 1 (#5), 2 (#1) **Ankle plantar flexors (*p* = 0.01)** Pre: 1 (#1), 2 (#1), 3 (#6), 4 (#1) Post: 1 (#2), 2 (#5), 3 (#2), 4 (#1)	**Shoulder abduction (*p* = 0.02)** Pre: 1 (#2), 1+ (#3), 2 (#4) Post: 1 (#5), 1+ (#) **Elbow flexors (*p* = 0.02)** Pre: 1 (#3), 1+ (#4), 2 (#2) Post: 0 (#1), 1 (#7), 2 (#1) **Forearm pronators (*p* = 0.04)** Pre: 1 (#3), 1+ (#1), 2 (#4), 3 (#1) Post: 0 (#1), 1 (#4), 1+ (#3), 2 (#1) **Hip adductors (*p* = 0.05)** Pre: 0 (#1), 1 (#3), 1+ (#1), 2 (#3), 3 (#1) Post: 0 (#2), 1 (#3), 1+ (#3), 1 (#1) **Knee extensors (*p* = 0.01)** Pre: 0 (#1), 1 (#3), 1+ (#3), 2 (#2) Post: 0 (#5), 1 (#2), 1+ (#2) **Ankle plantar flexors (*p* = 0.10)** Pre: 2 (#2), 3 (#3), 4 (#4) Post: 1+ (#1), 2 (#2), 3 (#3), 4 (#3)
					30 min music + 30 to 45 min conventional therapy, 1x/day, 3 days		Shoulder abduction: *p* = 0.87 Elbow flexors: *p* = 0.39 Forearm pronators: *p* = 0.56 Hip adductors: *p* = 0.32 Knee extensors: *p* = 0.74 Ankle plantar flexors: *p* = 0.13	
Kvam (1997)	CP (*n* = 12, 9/3)	40.5	Music chair	NWMT	Vibroacoustic therapy (*n* = 6)	Only music (*n* = 6)	No significant differences based on treatment (no statistical results) None was regarded worse after treatment. No tendency was observed for different muscle groups.	
					25 to 30 min, 2x/week, 8 weeks			
Puggina & Da Silva (2015)	Coma, VS or sedated (*n* = 76, 56/20)	42.5 (19)	Music between 60 and 70 dB, selected by family members, patient’s preferences	ENG (ratio)	Exp 1: Music (*n* = 30) Exp 2: Messages (*n* = 26)	Silence (*n* = 20)	**Music** Relaxation: 4:25 Tension: 12:75 No alteration: 0:0 **Messages** Relaxation: 3:23 Tension: 10:76.9 No alteration: 0:0	**Silence** Relaxation: 7:70 Tension: 2:20 No alteration: 1:10
					2 to 4 min, 2 sessions		*p* = 0.019 (only data of participants in coma/vegetative state)	
Scartelli (1982)	CP (*n* = 6, 3/3)	23–33	Music 45–60 dB, -stereo cassette unit	EMG (µV)	Sedative music-assisted EMG biofeedback relaxation training (*n* = 3)	Only EMG biofeedback relaxation training (*n* = 3)	Decrease of 65% in finger extensor muscle activity	Decrease of 32.5% in finger extensor muscle activity
					20 min, 3x/week, 5 weeks			
Wong, Mak & Mok (2018)	Stroke, CP and TBI (*n* = 40, 21/19)	40 (15.9) 37 (16.3)	2 types of relaxing music—portable music player	MAS (/5)	Mozart K.448 (*n* = 20)	Smooth, soft relaxing music (*n* = 20)	**Left elbow** Pre: 1.85 Post: 1.35 **Right elbow** Pre: 1.65 Post: 1.05 **Left knee:** Pre: 1.75 Post: 1.30 **Right knee:** Pre: 1.75 Post: 1.25	**Left elbow** Pre: 1.87 Post: 1.70 **Right elbow** Pre: 1.75 Post: 1.17 **Left knee:** Pre: 2.25 Post: 1.95 **Right knee:** Pre: 2.15 Post: 1.72
					8 min, 3x/week, 8 weeks		Left elbow: *p* = 0.161 Right elbow: *p* = 0.260 Left knee: *p* = 0.740 Right knee: *p* = 0.917	

**Notes.**

Abbreviations *n* number of m men w woman SD standard deviation CP cerebral palsy VS vegetative state TBI traumatic brain injury dB decibels CCHQ comfort and care hypertonicity questionnaire MAS modified Ashworth scale NWMT Nic Waals muscle test ENG electroneuromyography EMG electromyography µV microvolts x times exp experimental pre pre-treatment post post-treatment # amount of freq frequency amp amplitude

To facilitate the quantitative interpretation of the randomized controlled trials, forest plots were created using RevMan 5.3 (Review Manager (RevMan), 2014. Version 5.3. Copenhagen: The Nordic Cochrane Centre). Since a great amount of heterogeneity was present in the outcome measures and assessed muscles resulting in difficulties presenting each measure and muscle separately, a generalized outcome measure “muscle performance” was created. Multiple outcome measures concerning muscle tone within one study were collectively estimated as one outcome measure to ensure an accurate and general image of muscle performance. Either the mean of different muscles or the mean of several muscle tone outcome measures were used to calculate muscle performance. Combining outcome measures should only be allowed when similar responsiveness has been reported (Puhan et al., 2006), which is the case in this study since the combined outcome measurement were assessed with the same assessment tool. The recalculated mean differences and standard deviations were inserted in the RevMan 5.3 template. When the necessary data was not available, authors were contacted to complete the data form. If authors did not respond, missing data were manually calculated using the RevMan 5.3 calculator, if possible and if necessary. To calculate pooled effect sizes, inverse variance was used as statistical method, a random effects model was used as analysis model and standardized mean differences (SMD) were calculated as the effect measure. Heterogeneity between the studies was assessed using *I*^2^ statistics (Higgins et al., 2019; Higgins & Thompson, 2002). Cochrane guidelines were used to interpret the heterogeneity: 0–40% (might not be important), 30–60% (may represent moderate heterogeneity), 50–90% (may represent substantial heterogeneity), and 75–100% (considerable heterogeneity) (Higgins et al., 2019). Effect sizes were categorized as a standard mean effect size of 0 which represented no change, 0.2 representing a small effect, 0.5 representing a medium effect and 0.8 representing a large effect (Cohen, 1988). Based on the standardized mean differences extracted from the meta-analysis, a Spearman’s correlation analysis was performed with the amount of therapy time. Confidence intervals (CI) were set to 95%.

## Results

### Study selection

**Figure 1:**
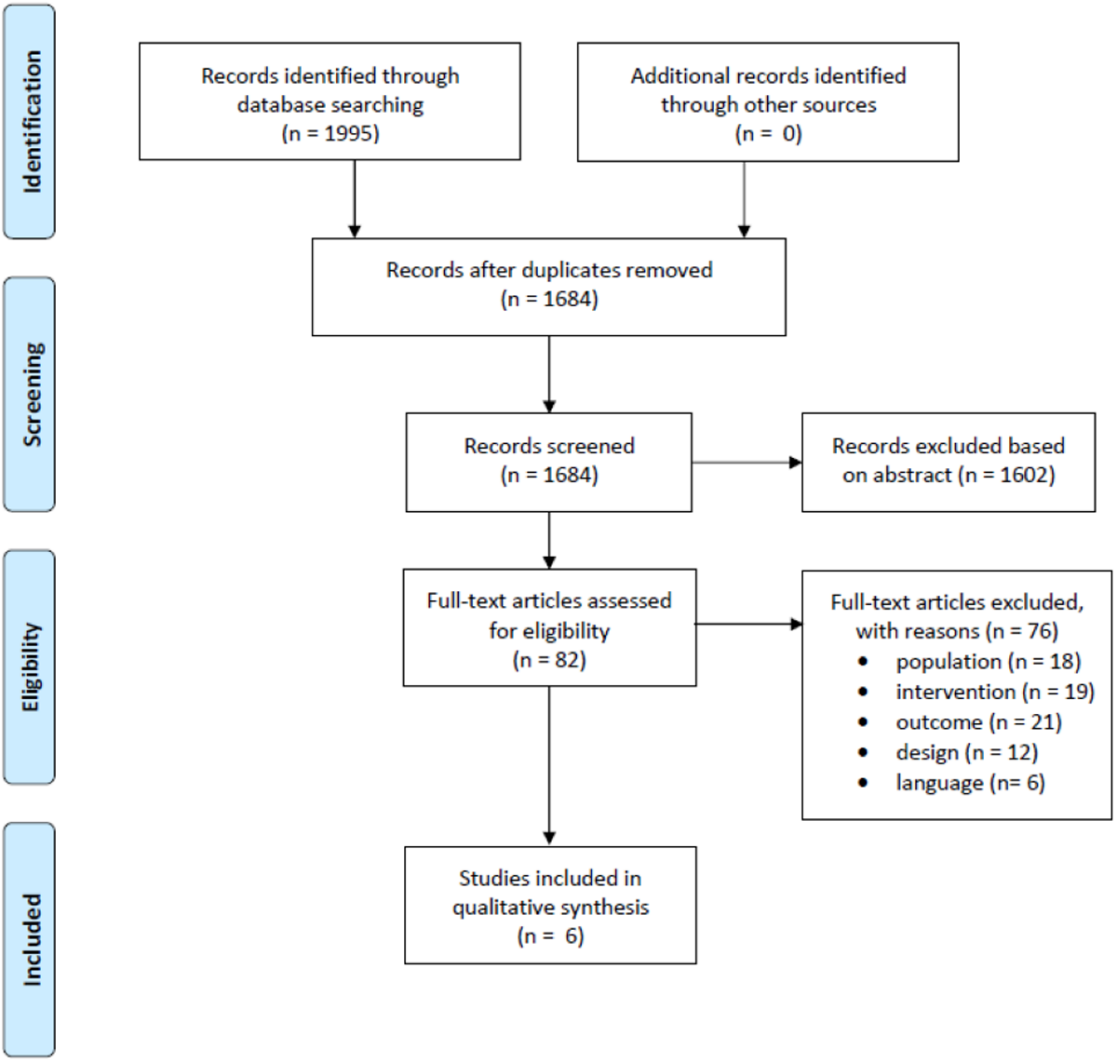
PRISMA flow chart of the included studies.

Of the 1995 studies obtained from all databases, 6 studies met all inclusion criteria. The study selection process is depicted in Fig. 1. Concerning the quality assessment, a median score of 6.5 was observed with a maximum of eight and minimum of three (see Table 2). In total, four studies had a high methodological quality, while the other two had a fair and poor quality. Most studies did not meet the criteria of blinding of participants and therapist as this does not seem possible with respect to treatment. Listening to music is quite apparent for both patient and therapist compared to a placebo or no therapy. In addition, only two studies reported variability measures which makes quantitative analysis difficult.

**Table 2:** Risk of bias assessment with PEDro scale.

**Author (Year)**	**Eligibility**	**Random allocation**	**Concealed allocation**	**Baseline comparability**	**Blinding subjects**	**Blinding therapist**	**Blinding assessors**	**Adequate follow-up**	**Intention-to-treat analysis**	**Between-group statistical comparison**	**Point measures and measures of variability**	**Score**
Ben-Pazi et al. (2018)	Yes	Yes	No	Yes	Yes	No	Yes	No	Yes	Yes	No	**6**
Jeba & Joshi (2016)	Yes	Yes	Yes	Yes	No	No	No	Yes	Yes	Yes	Yes	**7**
Kvam (1997)	No	Yes	No	No	No	No	Yes	Yes	No	Yes	No	**4**
Puggina & Da Silva (2015)	Yes	Yes	Yes	Yes	Yes	No	Yes	Yes	No	Yes	Yes	**8**
Scartelli (1982)	No	Yes	No	No	No	No	No	Yes	Yes	No	No	**3**
Wong, Mak & Mok (2018)	Yes	Yes	Yes	Yes	Yes	No	No	No	No	Yes	No	**5**

### Study characteristics

In total, data from 171 patients (50 females, 102 males, 19 unknown), of which 30 were diagnosed with stroke (Jeba & Joshi, 2016; Wong, Mak & Mok, 2018), 61 with cerebral palsy (Ben-Pazi et al., 2018; Kvam, 1997; Scartelli, 1982; Wong, Mak & Mok, 2018), 4 with traumatic brain injury (Wong, Mak & Mok, 2018) and 76 patients in a decreased conscious state (Puggina & Da Silva, 2015) were included in this study (Table 1). One hundred and four participants were allocated to the experimental group receiving MLI, while 67 patients were in the control group. The age of the examined subjects ranged between 7 and 53 years.

There was a great variety in outcomes measures used to assess hypertonia. Two studies assessed muscle tone clinically by means of the Modified Ashworth Scale (MAS) (Jeba & Joshi, 2016; Wong, Mak & Mok, 2018), while the other studies used the Care and Comfort Hypertonicity Questionnaire (Ben-Pazi et al., 2018) or Nic Waals muscle test (Kvam, 1997). Hypertonia and muscle activity was biomechanically assessed by either electroneuromyography (ENG) (Puggina & Da Silva, 2015) or EMG (Scartelli, 1982).

Three studies used MLI as an additional technique during rehabilitation—vibroacoustic (Kvam, 1997), biofeedback relaxation training (Scartelli, 1982) and conventional neurorehabilitation (Jeba & Joshi, 2016). Vibroaccoustic therapy was executed with a relaxation chair with built-in loudspeakers, the loudspeakers are able to produce low frequency acoustic vibrations and music simultaneously. Biofeedback relaxation training was executed with a Cyborg EMG biofeedback unit (Model #EMG J33), feedback of the muscular activity was seen on screen and by a clicking sound to keep patients aware of the acceptable level of muscle activity. Conventional neurorehabilitation consisted of inhibitory techniques for hypertonia, facilitatory techniques for antagonistic muscles, weight bearing exercises, stretching and rhythmic rotations. Other studies used music listening in comparison to no music (Puggina & Da Silva, 2015) or different music sounds (Ben-Pazi et al., 2018; Wong, Mak & Mok, 2018). The amount of interventions ranged from two to 24 sessions, while therapy time ranged from two minutes to 30 min. Four studies (Ben-Pazi et al., 2018; Kvam, 1997; Scartelli, 1982; Wong, Mak & Mok, 2018) investigated long-term effects, ≥4 weeks of therapy, while two studies (Jeba & Joshi, 2016; Puggina & Da Silva, 2015) examined short-term effects, <1 week of therapy.

The type of music was either predetermined by the study protocol (Jeba & Joshi, 2016; Kvam, 1997; Scartelli, 1982; Wong, Mak & Mok, 2018) or based on the participant’s preferences (Ben-Pazi et al., 2018; Puggina & Da Silva, 2015). Predetermined music was selected by the researchers and consisted of the following musical pieces:

1. Scartelli (1982): “The Gift” and “Grandfather’s Story” in The Red Pony by Aaron Copland (Columbia Y31016); “Aspen” in Captured Angel by Dan Fogelberg (Epic PE 33499); “Lullabye” in Children of Sanchez by Chuck Mangione (A & M SP 6700); and “Trois Gymnopedies” in Blood, Sweat, and Tears by Eric Satie (Columbia CS 9720);
2. Jeba & Joshi (2016): Eastern classical instrumental pieces which were described as relaxing;
3. Wong, Mak & Mok (2018) compared to musical pieces: The Mozart K.448 musical piece was selected for the experimental group compared to a relaxing melody which was randomly searched on the internet using keywords “relaxing music, 8 min”. The chosen piece was composed by Michael in 2011 and was played on an electronic piano (https://www.youtube.com/watch?v=MAjXiyKPBu8).

Some studies did not mention the music selection process or music pieces used (Kvam, 1997). The two studies who allowed music based on the patient’s preferences selected the music as follows:

1. Puggina & Da Silva (2015): Family members were asked to choose the patient’s (with decreased conscious state) favorite music style from a predetermined list with 98 songs. Eight different musical styles were represented in this list, i.e., Brazilian country music, samba, international music, movie themes, new age, classical, and gospel.
2. Ben-Pazi et al. (2018) compared two musical pieces: The control group received 4 to 5 audio tracks including music or nature sounds according to the child’s or parent’s preferences, while the experimental group also received music or nature sound to the child’s or parent’s preferences with fixed sound frequencies embedded in the musical piece.

### Synthesis of results

Half of the included studies concluded that MLI is effective in influencing muscle tone (Ben-Pazi et al., 2018; Puggina & Da Silva, 2015; Scartelli, 1982), while the others found no significant between-group differences between experimental and control therapy (Jeba & Joshi, 2016; Kvam, 1997; Wong, Mak & Mok, 2018). Detailed results of the individual studies can be found in Table 1 and as Supplementary Material 3. Although the studies did not find any significant between-group differences, the findings support the use of MLI in reducing hypertonia. First, Wong, Mak & Mok (2018) concluded that both Mozart K.448 and general relaxing music were able to reduce the spasticity levels after eight weeks, no differences were found between-groups (two types of music). Second, Jeba & Joshi (2016) did not find any significant between-group differences (conventional therapy vs. conventional therapy with music), yet concluded that the clinical impressions suggested supportive effects of MLI on spasticity. They found decreased spasticity levels after only three sessions within one week in the ankle plantar flexors (*p* = 0.01) and hip adductors (*p* = 0.04) which were not found with conventional therapy alone. Kvam (1997) found no significant differences between music plus vibroacoustic therapy compared to music alone, yet both groups significantly improved after eight weeks.

**Figure 2:**
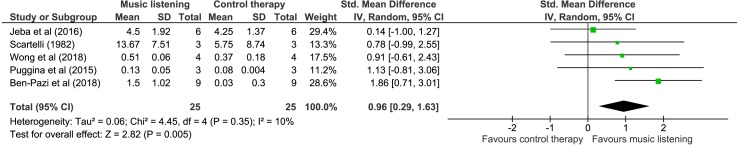
Effectiveness of music listening on muscle performance: forest plot.

In total, five of the six studies and 133 of the 171 patients were included in the quantitative analysis since Kvam (1997) did not provide any data or statistical analysis (only descriptive). The parameter muscle performance was used as a combined outcome measures for different muscles in the study of Jeba & Joshi (2016) and Wong, Mak & Mok (2018), and muscle tension assessments in the study of Puggina & Da Silva (2015). The entire population dataset of Scartelli (1982) and Ben-Pazi et al. (2018) used only one outcome measure or muscle, which allowed calculation of mean (and SD) muscle tone between participants. The analysis shows that there was a large treatment effect of MLI on muscle performance (SMD 0.96, 95% CI [0.29–1.63]) as depicted in Fig. 2. The level of heterogeneity was considered not important (*I*^2^ = 10%). No correlation between the effectiveness of MLI and amount of therapy was found (*r* = 0.30, *p* = 0.62).

## Discussion

### Synthesis of results

The aim of this study was to investigate the effectiveness of MLI on hypertonia in neurological patients. We reviewed a total of six randomized controlled trials that provided information of 171 neurologically impaired patients after MLI. The overall risk of bias of the included studies was moderate to low, three studies had a high methodological quality, while two had a fair and one a poor quality. Although not all studies reported between-group differences, all reported improvement in muscle tone over time. Although it is difficult to differentiate these results from natural recovery as no study provided follow-up data after the treatment period, it is reasonable to assume that spasticity tends to get worse if left untreated. A general conclusion of the quantitative analysis suggests that the treatment effect after MLI on hypertonia in neurologically impaired patients was large (SMD 0.96, 95% CI [0.29–1.63]), with a comparatively low level of heterogeneity to declare (*I*^2^ = 10%). Yet, no correlation was found with the amount of therapy which was probably due to the low amount of studies included in the analysis.

### Pathology-dependent effectiveness

Four different pathologies were included in this review, patients with cerebral palsy, stroke, traumatic brain injury and decreased conscious state. No clear distinction in treatment effect could be made between the different pathologies. The pathophysiology of these disorders are related to upper motor neuron involvement and all of them had signs of pyramidal hypertonia. None of the included participants had extrapyramidal hypertonia or rigidity, which is a specific characteristic of Parkinson’s disease. Nevertheless, sound-based interventions are a major part in the rehabilitation of patients with Parkinson’s disease. The pathophysiology of these patients is related to degeneration of the substantia nigra, located in the basal ganglia (Galvan & Wichmann, 2008). Sound-based interventions such as auditory stimulation or cueing is used as a technique to bypass the involvement of the basal ganglia which enables the control of automatic movements in the premotor cortex, and does therefore not necessary influence muscle tone (Sarma et al., 2012). Current treatment options for extrapyramidal hypertonia are mainly pharmaceuticals or mobilization techniques. This led us to believe that maybe not all neurologically impaired patients benefit from MLI for reducing hypertonia and that the type of hypertonia is probably extremely important in obtaining the desired treatment effects. However, these results bring to light a clear gap in the literature—no studies, to our knowledge, have investigated the effect of MLI on extrapyramidal hypertonia or rigidity.

### Assessment of spasticity

Assessment of pyramidal hypertonia or spasticity is mainly performed based on clinical outcome measures such as the (Modified) Ashworth scale, (Modified) Tardieu scale or spasm frequency scales (Biering-Sorensen, Nielsen & Klinge, 2006; Rekand, 2010). However, since spasticity scales are dependent on the therapist’s experience and judgement, they are deemed invalid and unreliable (Fleuren et al., 2010; Kumar, Pandyan & Sharma, 2006). Therefore, the authors suggest to stop using these subjective outcome measures and include more promising techniques such as surface EMG (Fleuren et al., 2010; Kumar, Pandyan & Sharma, 2006) or considering the impact on functioning and activities of daily living (Rekand, 2010). Although there is a definite need for simple clinical outcome tools, accurate and reliable assessment of spasticity is mainly performed by combining biomechanical with electrophysiological techniques (Biering-Sorensen, Nielsen & Klinge, 2006; Kim et al., 2005; Kumar, Pandyan & Sharma, 2006). However, in this current review the majority of studies used clinical outcome measures, while only two used electrophysiological assessment tools. ENG analysis was performed in patients with a decreased conscious state and EMG was assessed in patients with cerebral palsy. The limited amount of research in these two population groups and the lack of evidence in others e.g., stroke or traumatic brain injury represents the need for further research to adequately quantify the effectiveness of MLI on spasticity.

### Music as an intervention

In this review music was either supportive of another rehabilitation technique (Ben-Pazi et al., 2018; Jeba & Joshi, 2016; Kvam, 1997; Scartelli, 1982) or a stand-alone treatment (Puggina & Da Silva, 2015; Wong, Mak & Mok, 2018). Treatment effects were of greater magnitude for the latter (SMD 0.91; 1.13) compared to executed as supportive treatment (SMD 0.78; 0.14), except for Ben-Pazi et al., who found the greatest effects (SMD 1.86) comparing gamma stimulation to only music suggesting the importance of specific frequency ranges. However, the general tendency of the results is not so surprising, since treatment effects in music as a supportive therapy are probably generated by the primary therapy. Therefore differences in treatment effect between both groups will be smaller as to comparing treatment effects which are solely generated by music. During these type of listening sessions the focus on the music is much higher than during exercises. The main focus of the patient during conventional therapy is accurate execution of the performed task which might result in decreased attention being directed to the music. On the other hand, if the patient is actively listening to the music, they are executing a dual-task. Deficits in divided attention and dual-tasks have been widely reported in neurologically impaired patients (Mathias & Wheaton, 2007; Yang et al., 2007; Yogev et al., 2005). It might be that, although it is important to train dual-tasks, decreased treatment effects were a result of divided attention in comparison to the single-task where patients only had to focus on the music. Although divided attention between motor task and music generates smaller treatment effects, they are still present. Clinically, this would suggest that MLI can be used either as background music during rehabilitation (dual-task) or during rest (single-task) to induce muscle relaxation.

### Characteristics of music

Self-selected music seems to affect physiological parameters such as muscle activity, heart and respiration rate in a different way than not self-selected music (Blood & Zatorre, 2001). Different types of music, such as jazz, rock, country or blues, affect specific brain regions and result in various effects in EEG waves (Sun et al., 2013). These music preferences are generally discriminated in the frontal cortex of the brain, which seems highly dependent on frequency (gamma) bands (Pan et al., 2013; Sammler et al., 2007). As spasticity is a result of an upper motor neuron lesion, located in the cortex or brain stem, inducing changes in brain activity could therefore result in differences in muscle tone. In this review, there were two studies using patient’s preferred music, in children with cerebral palsy and adults with decreased conscious state (Ben-Pazi et al., 2018; Puggina & Da Silva, 2015). These studies reported the largest treatment effects of all the included studies. One study compared two different forms of relaxation music (fixed protocol frequency embedded in preferred music compared only preferred music) in children with CP and found a significant decrease on the Care and Comfort Hypertonicity Questionnaire (SMD 1.86, 95% CI [0.71–3.01]) (Ben-Pazi et al., 2018). Listening to music in patients with a decreased conscious state resulted in greater muscle tension (*p* = 0.019) and non-significant improvements in muscle performance (SMD 1.13, 95% CI [−0.81 to 3.06]) (Puggina & Da Silva, 2015). It seems that music preferences are of major importance when selecting songs when aiming to influence muscle tone. Studies have shown that music preferences, inherent to musical genre, are dependent on three music attributes (Fricke et al., 2018; Greenberg et al., 2016): (1) arousal: tense and strong; (2) valence: amusing but not depressing; and (3) depth: complex, deep and intelligent. These music dimensions are, in its turn, related to personality traits, social connotation, race, cognitive abilities and self-views (Marshall & Naumann, 2018; Rentfrow, Goldberg & Levitin, 2011; Rentfrow & Gosling, 2003)

### Music listening in neurologically impaired patients

The majority of these studies investigating brain activity during music listening are performed on healthy subjects. Normal music listening involves several cortical areas extending well beyond the primary auditory cortex, such as the corpus callosum, superior-temporal plane and middle/inferior temporal gyri (Stewart et al., 2006). Yet, neurologically impaired patients have acquired lesions in the brain which could influence music listening. Damage of these areas can lead to deficits in the processing of pitch, timbre, timing and emotional response (Stewart et al., 2006). Since the majority of these areas is located in the right hemisphere, patients with left hemiplegia are more predisposed for musical deficits. Moreover, it seems possible to use pathology-specific music, by considering favorable frequencies of several brain structures (Miller et al., 2017). For example, thalamic lesion would benefit most from musical frequencies at 2,300, 2,349 and 2,400 Hz in note D and in the 7th octave (Miller et al., 2017). This bio-guided model of brain music was also adopted by Miller (2011), who found that altering several music parameters can help in specifically targeting stress, ADHD, aging and pain (Miller, 2011). Therefore it might be interesting not to only incorporate a patient’s preferences when selecting music, targeting the lesion locations may be of additional value during recovery. However, more research regarding this bio-guided model for MLI is necessary.

### Directions for future research and clinical practice

Although results of this study suggest that MLI is able to induce muscle relaxation, the limited number of studies included prevents the implementation in clinical practice. The authors suggest that new studies with larger populations are needed to verify these findings. In addition, future studies should include objective measurement tools when assessing hypertonia as a primary outcome, while also examining the musical characteristics and functional tasks leading to the best outcomes.

### Limitations

There are a few limitations of this review that should be acknowledged. First, during the systematic literature search, only studies written in Dutch, English, German, French or Spanish were included. It is therefore possible relevant studies and important information was missed during the search process. Second, although the search strategy was elaborate, some caution for these proposed recommendations is required since conclusions were based on the results of only five studies (included in meta-analysis). Third, due to lack of standard deviations or statistical data provided in the articles or after contacting authors, we decided to create the outcome measures “muscle performance” which was the mean muscle tone of several individual muscles or various tension outcome measures. Although combining outcome measures with similar responsiveness has been described to be an effective method, including the raw data would be more statistically accurate. However, by adopting this new outcome measure, quantitative analysis could be performed which was otherwise not possible.

## Conclusions

Quantitative analysis of the results in this review suggest that MLI are able to increased muscle relaxation in neurologically impaired patients, although one study reported increased muscle activity. MLI can be used as either background music during rehabilitation (dual-task) or during rest (single-task). In addition, musical preferences seem to play a major role in the observed treatment effect. We therefore advice using patient’s preferred music when selecting songs. However, several gaps were found in the literature which necessitates further research. First of all, effectiveness of music listening was only examined in pyramidal hypertonia (spasticity) and no research was found on extrapyramidal hypertonia (rigidity). Second, a great amount of variety was present in the use of spasticity assessment tools. Only a limited amount of research has been performed with adequately quantifiable spasticity measurements in neurological patients. In conclusion, music listening and processing requires several cortical brain areas which might be affected after diagnosis. It might therefore be of interest to further explore a bio-guided model in MLI for these patients.

##  Supplemental Information

10.7717/peerj.8228/supp-1 Supplemental Information 1 PRISMA Checklist

10.7717/peerj.8228/supp-2 Supplemental Information 2 Search Strategy

10.7717/peerj.8228/supp-3 Supplemental Information 3 Raw Data

10.7717/peerj.8228/supp-4 Supplemental Information 4 Rationale and Contributions

## Additional Information and Declarations

### Data Availability

The following information was supplied regarding data availability: Most of the raw data is available in Table 1. The remaining data is available as Supplemental File.
