# Supplementary material for: Effect of music listening on hypertonia in neurologically impaired patients—systematic review
Source: PeerJ. 2019 Dec 19;7:e8228. doi: 10.7717/peerj.8228 (PMC6925946; doi:10.7717/peerj.8228)
Supplement: Supplemental Information 1 [file peerj-07-8228-s001.docx]

| **Section/topic** | **#** | **Checklist item** | **Reported on page #** |
| --- | --- | --- | --- |
| **TITLE** | | |  |
| Title | 1 | Identify the report as a systematic review, meta-analysis, or both. | 1 |
|  |  | *Quote from manuscript:* “Effect of music listening on hypertonia in neurologically impaired patients - systematic review” |  |
| **ABSTRACT** | | |  |
| Structured summary | 2 | Provide a structured summary including, as applicable: background; objectives; data sources; study eligibility criteria, participants, and interventions; study appraisal and synthesis methods; results; limitations; conclusions and implications of key findings; systematic review registration number. | 2 |
|  |  | *Quote from manuscript:*  “**Background**: As music listening is able to induce self-perceived and physiological signs of relaxation, it might be an interesting tool to induce muscle relaxation in patients with hypertonia. To this date effective non-pharmacological rehabilitation strategies to treat hypertonia in neurologically impaired patients in lacking. Therefore the aim is to investigate the effectiveness of music listening on muscle activity and relaxation.  **Methodology:** The search strategy was performed by the PRISMA guidelines and registered in the PROSPERO database (no. 42019128511). Seven databases were systematically searched until March 2019. Six of the 1684 studies met the eligibility criteria and were included in this review. Risk of bias was assessed by the PEDro scale. In total 171 patients with a variety of neurological condition were included assessing hypertonia with both clinically and biomechanical measures.  **Results:** The analysis showed that there was a large treatment effect of music listening on muscle performance (SMD 0.96, 95% CI 0.29 to 1.63, I²= 10%, Z=2.82, p=0.005). Music can be used as either background music during rehabilitation (dual-task) or during rest (single-task) and musical preferences seem to play a major role in the observed treatment effect.  **Conclusions:** Although music listening is able to induce muscle relaxation, several gaps in the available literature were acknowledged. Future research is in need of an accurate and objective assessment of hypertonia.” |  |
| **INTRODUCTION** | | |  |
| Rationale | 3 | Describe the rationale for the review in the context of what is already known. | 3 |
|  |  | *Quote from manuscript:*  “However, high-quality evidence for supporting the effectiveness of these rehabilitation techniques is lacking (Amatya, Khan, La Mantia, Demetrios, & Wade, 2013; Khan, Amatya, Bensmail, & Yelnik, 2017; Nair & Marsden, 2014). A low quality for evidence was found for rehabilitation programs, electrical stimulation, physical activity programs, vibration therapy, stretching and passive movement in neurological patients (Khan et al., 2017). There is a clear need for effective non-pharmacological rehabilitation strategies to treat spasticity. IListening to music activates cortical and paralimbic areas related to neural systems of reward and emotion (Blood & Zatorre, 2001), which makes music an intervention which can be rewarding and motivating and at the same time regulate emotions, arousal and cognitive functions (Sarkamo, 2018). So music interventions might be a good multi-modal treatment option for inducing muscle relaxation in neurological patients with hypertonia, and might lead to a better therapy compliance by its enjoyable character. “ |  |
| Objectives | 4 | Provide an explicit statement of questions being addressed with reference to participants, interventions, comparisons, outcomes, and study design (PICOS). | 4 |
|  |  | *Quote from manuscript:*  “The aim of this study is to investigate if music listening interventions (MLI) are an effective tool to decrease muscle tension in neurologically impaired patients suffering from hypertonia. As listening to music is able to induce general relaxation in several patient populations, we hypothesize that relaxation on muscular level will be present” |  |
| **METHODS** | | |  |
| Protocol and registration | 5 | Indicate if a review protocol exists, if and where it can be accessed (e.g., Web address), and, if available, provide registration information including registration number. | 4 |
|  |  | *Quote from manuscript:*  “This review was conducted according to the Preferred Reporting Items for Systematic Review and Meta-Analysis Statement (PRISMA). The checklist can be found as supplementary material (Supplementary material 1) (Moher, Liberati, Tetzlaff, Altman, & Group, 2009). The study was registered in the PROSPERO database (CRD: no. 42019128511).” |  |
| Eligibility criteria | 6 | Specify study characteristics (e.g., PICOS, length of follow-up) and report characteristics (e.g., years considered, language, publication status) used as criteria for eligibility, giving rationale. | 4 |
|  |  | *Quote from manuscript:*  “Studies were included if they met the following criteria:  1) The study population included patients suffering from a neurological disorder which could result in hypertonia e.g. stroke, cerebral palsy, Parkinson’s disease, spinal cord injury, multiple sclerosis, etc.  2) Interventions had to include MLI  3) Muscle tone or activity had to be assessed after intervention. Both clinical and biomechanical analysis of muscle performance were included, e.g. modified Ashworth scale, motor assessment scale, electromyography (EMG), etc.  4) Studies had to be written in English, Dutch, German, French or Spanish.  5) Study design was a (randomized) clinical trial  Studies were excluded using the following criteria:  1) Music listening involving other sound-based interventions, e.g. RAS or auditory cueing;  2) Studies pertaining to active music interventions (i.e., singing, playing musical instruments);” |  |
| Information sources | 7 | Describe all information sources (e.g., databases with dates of coverage, contact with study authors to identify additional studies) in the search and date last searched. | 5-6 |
|  |  | *Quote from manuscript:*  “A systematic search strategy was conducted (TVC) using the electronic databases of PubMed, Web of Science, Cochrane Library, Science Direct, Scopus, ResearchGate and the Physiotherapy Evidence Database (PEDro)”. “The details of the final search strategy, performed in March 2019, can be found as Supplementary Material 2.” |  |
| Search | 8 | Present full electronic search strategy for at least one database, including any limits used, such that it could be repeated. | SM1 + 6 |
|  |  | *Quote from manuscript:*  “The details of the final search strategy, performed in March 2019, can be found as Supplementary Material 2.” |  |
| Study selection | 9 | State the process for selecting studies (i.e., screening, eligibility, included in systematic review, and, if applicable, included in the meta-analysis). | 5 |
|  |  | *Quote from manuscript:*  “The screening procedure was performed by three independent researchers (T.V.C., J.O. and K.D.). To collect potentially relevant studies, eligibility was screened based on title and abstract. Full texts were retrieved and evaluated based on the a-priori provided inclusion and exclusion criteria. Afterwards full texts were gathered and evaluated on the previously set inclusion criteria. Reference lists were manually screened to identify additional relevant studies. Discrepancies were discussed with a third independent person (either J.O or K.D., depending on the allocated studies).” |  |
| Data collection process | 10 | Describe method of data extraction from reports (e.g., piloted forms, independently, in duplicate) and any processes for obtaining and confirming data from investigators. | 5-6 |
|  |  | *Quote from manuscript:*  Extracted data consisted of subject characteristics (age, gender, pathology), outcome measures, type of music or apparatus, intervention and results (see Table 1). Results were mostly described as a difference between groups based on intervention |  |
| Data items | 11 | List and define all variables for which data were sought (e.g., PICOS, funding sources) and any assumptions and simplifications made. | 4,6 |
|  |  | *Quote from manuscript:*  “Since a great amount of heterogeneity was present in the outcome measures and assessed muscles resulting in difficulties presenting each measure and muscle separately, a generalized outcome measure “muscle performance” was created. Multiple outcome measures concerning muscle tone within one study were collectively estimated as one outcome measure to ensure an accurate and general image of muscle performance. Either the mean of different muscles or the mean of several muscle tone outcome measures were used to calculate muscle performance. Combining outcome measures should only be allowed when similar responsiveness has been reported (Puhan, Soesilo, Guyatt, & Schunemann, 2006), which is the case in this study since the combined outcome measurement were assessed with the same assessment tool.” |  |
| Risk of bias in individual studies | 12 | Describe methods used for assessing risk of bias of individual studies (including specification of whether this was done at the study or outcome level), and how this information is to be used in any data synthesis. | 5 |
|  |  | *Quote from manuscript:*  “The risk of bias was assessed by two independent reviewers (T.V.C. and E.C.) by using the PEDro scale for randomized controlled trials (Maher, Sherrington, Herbert, Moseley, & Elkins, 2003). In case of uncertainty at any point during the scoring process, consensus was sought by a third reviewer (J.O.). The PEDro scale assesses eleven items such as random allocation of the subjects, concealed allocation and blinding of therapists and assessors (Maher et al., 2003). The total PEDro score can be divided into three sections; high quality = PEDro score 6-10, fair quality = PEDro score 4-5 and poor quality = PEDro score < 3.” |  |
| Summary measures | 13 | State the principal summary measures (e.g., risk ratio, difference in means). | 5-6 |
|  |  | *Quote from manuscript:*  “Since a great amount of heterogeneity was present in the outcome measures and assessed muscles resulting in difficulties presenting each measure and muscle separately, a generalized outcome measure “muscle performance” was created.” |  |
| Synthesis of results | 14 | Describe the methods of handling data and combining results of studies, if done, including measures of consistency (e.g., I^2^) for each meta-analysis. | 6-7 |
|  |  | *Quote from manuscript:*  “The recalculated mean differences and standard deviations were inserted in the RevMan 5.3 template. When the necessary data was not available, authors were contacted to complete the data form. If authors did not respond, missing data were manually calculated using the RevMan 5.3 calculator, if possible and if necessary. To calculate pooled effect sizes, inverse variance was used as statistical method, a random effects model was used as analysis model and standardized mean differences (SMD) were calculated as the effect measure. Heterogeneity between the studies was assessed using I² statistics (J. Higgins & Green, 2011; J. P. Higgins & Thompson, 2002). Cochrane guidelines were used to interpret the heterogeneity: 0-40% (might not be important), 30-60% (may represent moderate heterogeneity), 50-90% (may represent substantial heterogeneity), and 75-100% (considerable heterogeneity) (J. Higgins & Green, 2011). Effect sizes were categorized as a standard mean effect size of 0 which represented no change, 0.2 representing a small effect, 0.5 representing a medium effect and 0.8 representing a large effect (Cohen, 1988). Based on the standardized mean differences extracted from the meta-analysis, a Spearman’s correlation analysis was performed with the amount of therapy time. Confidence intervals (CI) were set to 95%.” |  |

Page 1 of 2

| **Section/topic** | **#** | **Checklist item** | **Reported on page #** |
| --- | --- | --- | --- |
| Risk of bias across studies | 15 | Specify any assessment of risk of bias that may affect the cumulative evidence (e.g., publication bias, selective reporting within studies). | n.a. |
| Additional analyses | 16 | Describe methods of additional analyses (e.g., sensitivity or subgroup analyses, meta-regression), if done, indicating which were pre-specified. | 7 |
|  |  | *Quote from manuscript* :  “Based on the standardized mean differences extracted from the meta-analysis, a Spearman’s correlation analysis was performed with the amount of therapy time.” |  |
| **RESULTS** | | |  |
| Study selection | 17 | Give numbers of studies screened, assessed for eligibility, and included in the review, with reasons for exclusions at each stage, ideally with a flow diagram. | 7 |
|  |  | *Quote from manuscript:*  “Of the 1995 studies obtained from all databases, 6 studies met all inclusion criteria. The study selection process is depicted in Figure 1.” |  |
| Study characteristics | 18 | For each study, present characteristics for which data were extracted (e.g., study size, PICOS, follow-up period) and provide the citations. | 7-8 |
|  |  | *Quote from manuscript:*  “In total, data from 171 patients (50 females, 102 males, 19 unknown), of which 30 were diagnosed with stroke (Jeba & Joshi, 2016; Wong, Mak, & Mok, 2018), 61 with cerebral palsy (Ben-Pazi et al., 2018; Kvam, 1997; Scartelli, 1982; Wong et al., 2018), 4 with traumatic brain injury (Wong et al., 2018) and 76 patients in a decreased conscious state (Puggina & da Silva, 2015) were included in this study (Table 1). … “ |  |
| Risk of bias within studies | 19 | Present data on risk of bias of each study and, if available, any outcome level assessment (see item 12). | 7 |
|  |  | *Quote from manuscript:*  “Concerning the quality assessment, a median score of 6.5 was observed with a maximum of eight and minimum of three (see Table 2). In total, four studies had a high methodological quality, while the other two had a fair and poor quality. Most studies did not meet the criteria of blinding of participants and therapist as this does not seem possible with respect to treatment.” |  |
| Results of individual studies | 20 | For all outcomes considered (benefits or harms), present, for each study: (a) simple summary data for each intervention group (b) effect estimates and confidence intervals, ideally with a forest plot. | Table 1 + SM3 + 9 |
|  |  | *Quote from manuscript:*  “Half of the included studies concluded that MLI is effective in influencing muscle tone (Ben-Pazi et al., 2018; Puggina & da Silva, 2015; Scartelli, 1982), while the others found no significant between-group differences between experimental and control therapy (Jeba & Joshi, 2016; Kvam, 1997; Wong et al., 2018). Detailed results of the individual studies can be found in Table 1 and as supplementary material 3.” |  |
| Synthesis of results | 21 | Present results of each meta-analysis done, including confidence intervals and measures of consistency. | Figure 2 + 10 |
|  |  | *Quote from manuscript:*  “The analysis shows that there was a large treatment effect of MLI on muscle performance (SMD 0.96, 95% CI 0.29 to 1.63) as depicted in Figure 2. The level of heterogeneity was considered not important (I²= 10%). No correlation between the effectiveness of MLI and amount of therapy was found (r=0.30, p=0.62).” |  |
| Risk of bias across studies | 22 | Present results of any assessment of risk of bias across studies (see Item 15). | Table 2 + 7 |
|  |  | *Quote from manuscript:*  “Concerning the quality assessment, a median score of 6.5 was observed with a maximum of eight and minimum of three (see Table 2). In total, four studies had a high methodological quality, while the other two had a fair and poor quality. Most studies did not meet the criteria of blinding of participants and therapist as this does not seem possible with respect to treatment.” |  |
| Additional analysis | 23 | Give results of additional analyses, if done (e.g., sensitivity or subgroup analyses, meta-regression [see Item 16]). | 10 |
|  |  | *Quote from manuscript:*  No correlation between the effectiveness of MLI and amount of therapy was found (r=0.30, p=0.62). |  |
| **DISCUSSION** | | |  |
| Summary of evidence | 24 | Summarize the main findings including the strength of evidence for each main outcome; consider their relevance to key groups (e.g., healthcare providers, users, and policy makers). | 11-14 |
|  |  | *Quote from manuscript*  “The aim of this study was to investigate the effectiveness of MLI on hypertonia in neurological patients. We reviewed a total of six randomized controlled trials that provided information of 171 neurologically impaired patients after MLI. The overall risk of bias of the included studies was moderate to low, three studies had a high methodological quality, while two had a fair and one a poor quality. Although not all studies reported between-group differences, all reported improvement in muscle tone over time. Although, it is difficult to differentiate these results from natural recovery as no study provided follow-up data after the treatment period, it is reasonable to assume that spasticity tends to get worse if left untreated. A general conclusion of the qualitative analysis suggests that the treatment effect after MLI on hypertonia in neurologically impaired patients was large (SMD 0.96, 95% CI 0.29 to 1.63), with a comparatively low level of heterogeneity to declare (I²=10%). Yet, no correlation was found with the amount of therapy which was probably due to the low amount of studies included in the analysis.” |  |
| Limitations | 25 | Discuss limitations at study and outcome level (e.g., risk of bias), and at review-level (e.g., incomplete retrieval of identified research, reporting bias). | 14 |
|  |  | *Quote from manuscript* :  “There are a few limitations of this review that should be acknowledged. First, during the systematic literature search, only studies written in Dutch, English, German, French or Spanish were included. It is therefore possible relevant studies and important information was missed during the search process. Second, although the search strategy was elaborate, some caution for these proposed recommendations is required since conclusions were based on the results of only five studies (included in meta-analysis). Third, due to lack of standard deviations or statistical data provided in the articles or after contacting authors, we decided to create the outcome measures “muscle performance” which was the mean muscle tone of several individual muscles or various tension outcome measures. Although combining outcome measures with similar responsiveness has been described to be an effective method, including the raw data would be more statistically accurate. However, by adopting this new outcome measure, qualitative analysis could be performed which was otherwise not possible. “ |  |
| Conclusions | 26 | Provide a general interpretation of the results in the context of other evidence, and implications for future research. | 15 |
|  |  | *Quote from manuscript* :  “Qualitative analysis of the results in this review suggest that MLI are able to increased muscle relaxation in neurologically impaired patients, although one study reported increased muscle activity. MLI can be used as either background music during rehabilitation (dual-task) or during rest (single-task). In addition, musical preferences seem to play a major role in the observed treatment effect. We therefore advice using patient’s preferred music when selecting songs. However, several gaps were found in the literature which necessitates further research. First of all, effectiveness of music listening was only examined in pyramidal hypertonia (spasticity) and no research was found on extrapyramidal hypertonia (rigidity). Second, a great amount of variety was present in the use of spasticity assessment tools. Only a limited amount of research has been performed with adequately quantifiable spasticity measurements in neurological patients. In conclusion, music listening and processing requires several cortical brain areas which might be affected after diagnosis. It might therefore be of interest to further explore a bio-guided model in MLI for these patients.” |  |
| **FUNDING** | | |  |
| Funding | 27 | Describe sources of funding for the systematic review and other support (e.g., supply of data); role of funders for the systematic review. | 15 |
|  |  | *Quote from manuscript* :  “Declaration of Sources of Funding. This research received no specific grant from any funding agency in the public, commercial, or not-for-profit sectors” |  |

*From:*  Moher D, Liberati A, Tetzlaff J, Altman DG, The PRISMA Group (2009). Preferred Reporting Items for Systematic Reviews and Meta-Analyses: The PRISMA Statement. PLoS Med 6(7): e1000097. doi:10.1371/journal.pmed1000097

For more information, visit: **www.prisma-statement.org**.

Page 2 of 2
