# Supplementary material for: Effect of music listening on hypertonia in neurologically impaired patients—systematic review
Source: PeerJ. 2019 Dec 19;7:e8228. doi: 10.7717/peerj.8228 (PMC6925946; doi:10.7717/peerj.8228)
Supplement: Supplemental Information 2 [file peerj-07-8228-s002.docx]

| **Database (n=studies)** | **Search strategy** |
| --- | --- |
| Web of Science (n=74) | (music) AND (spasticity OR "spastic" OR "muscle tone" OR "hypertonicity" OR "hypertonus" OR "hypertonic" OR hypertonia OR "muscle activity" OR electromyography) |
| Pubmed (n=204) |  |
| ScienceDirect (n=1397) |  |
| Scopus (n=266) |  |
| Cochrane Library (n=31) |  |
| PEDro (n=1) | music AND hypertonia |
| ResearchGate(n=22) | music and spasticity |
| **Supplementary material 1.** Search Strategy | |
