# Supplementary material for: Effect of music listening on hypertonia in neurologically impaired patients—systematic review
Source: PeerJ. 2019 Dec 19;7:e8228. doi: 10.7717/peerj.8228 (PMC6925946; doi:10.7717/peerj.8228)
Supplement: Supplemental Information 4 [file peerj-07-8228-s004.docx]

**Rationale for this systematic review:** A common symptom of upper motor neuron syndromes is hypertonia which is defined as an increased muscle tone. Spasticity-related interventions mainly consist of pharmacological and/or physio- and occupational therapeutic approaches (Katz, 1988; Nair & Marsden, 2014; Rekand, 2010; Thompson, Jarrett, Lockley, Marsden, & Stevenson, 2005). However, high-quality evidence for supporting the effectiveness of rehabilitation techniques on spasticity is lacking (Amatya, Khan, La Mantia, Demetrios, & Wade, 2013; Khan, Amatya, Bensmail, & Yelnik, 2017; Nair & Marsden, 2014). There is a clear need for effective non-pharmacological rehabilitation strategies to treat spasticity. Music interventions are gaining popularity in rehabilitation programs and are able to induce self-perceived and physiological outcomes of relaxation in healthy adults (Largo-Wight, O’Hara, & Chen, 2016; Madson & Silverman, 2010; Staum & Brotons, 2000; Stratton & Zalanowski, 1984), hospitalized (Krout, 2001; Sand-Jecklin & Emerson, 2010), burn (Tan, Yowler, Super, & Fratianne, 2010), psychiatric (M. H. Thaut, 1989), oncology (Cooper & Foster, 2008) and neurological patients (Sihvonen et al., 2017). As music is an intervention which can be rewarding and motivating and at the same time regulate emotions, arousal and cognitive functions (Sarkamo, 2018), it can be a good multi-modal treatment option for inducing muscle relaxation in neurological patients with hypertonia, and might lead to a better therapy compliance by its enjoyable character. The aim of this study is to investigate if music listening interventions (MLI) are an effective tool to decrease muscle tension in neurologically impaired patients suffering from hypertonia. As listening to music is able to induce general relaxation in several patient populations, we hypothesize that relaxation on muscular level will be present.

**Contribution of this study:**

To our knowledge, this is the first study qualitatively analyzing the available literature regarding the effectiveness of music listening intervention on muscle relaxation in neurologically impaired patients. Due to the lack of available studies, this review brought to light several gaps in the literature which necessitates further research, helping future researchers redirect their research questions. First of all, effectiveness of music listening was only examined in pyramidal hypertonia (spasticity) and no research was found on extrapyramidal hypertonia (rigidity). Second, a great amount of variety was present in the use of spasticity assessment tools. Only a limited amount of research has been performed with adequately quantifiable spasticity measurements in neurological patients. In conclusion, music listening and processing requires several cortical brain areas which might be affected after diagnosis. It might therefore be of interest to further explore a bio-guided model in MLI for these patients.
